# Biochemical and Expression Analyses Revealed the Involvement of Proanthocyanidins and/or Their Derivatives in Fiber Pigmentation of *Gossypium stocksii*

**DOI:** 10.3390/ijms23021008

**Published:** 2022-01-17

**Authors:** Yujie Sun, Diandian Zhang, Hongli Zheng, Yuqing Wu, Jun Mei, Liping Ke, Dongliang Yu, Yuqiang Sun

**Affiliations:** Plant Genomics & Molecular Improvement of Colored Fiber Laboratory, College of Life Sciences and Medicine, Zhejiang Sci-Tech University, Hangzhou 310018, China; sunyujie1111@126.com (Y.S.); devah0322@163.com (D.Z.); zhl0912ly@163.com (H.Z.); wuyuqing0104@163.com (Y.W.); 15110700054@fudan.edu.cn (J.M.); keliping@zstu.edu.cn (L.K.)

**Keywords:** wild cotton, brown fiber, proanthocyanidin, TT8, ANR

## Abstract

The wild cotton species *Gossypium stocksii* produces a brown fiber that provides a valuable resource for the color improvement of naturally colored cotton (NCC) fiber. However, the biochemical basis and molecular mechanism of its fiber pigmentation remain unclear. Herein, we analyzed the dynamics of proanthocyanidins (PAs) accumulation in developing the fiber of *G. stocksii*, which suggested a similar role of PAs and/or their derivatives in the fiber coloration of *G. stocksii*. In addition, comparative transcriptomics analyses revealed that the PA biosynthetic genes were expressed at higher levels and for a longer period in developing fibers of *G. stocksii *than* G. arboreum *(white fiber), and the transcription factors, such as TT8, possibly played crucial regulatory roles in regulating the PA branch genes. Moreover, we found that the anthocyanidin reductase (ANR) was expressed at a higher level than the leucoanthocyanidin reductases (LARs) and significantly upregulated during fiber elongation, suggesting a major role of ANR in PA synthesis in *G. stocksii *fiber. In summary, this work revealed the accumulation of PAs and the expression enhancement of PA biosynthetic genes in developing fibers of *G. stocksii*. We believe this work will help our understanding of the molecular mechanisms of cotton fiber coloration and further promote the future breeding of novel NCCs.

## 1. Introduction

Cotton fiber is the most important fabric material globally, with almost all the industrially used cotton coming from white cotton fiber (WCF). However, with the rising concern of environmental issues and the higher life quality of human beings, the interest in naturally colored cotton (NCC, *G. hirsutum*) fiber has continuously increased in the last decade. NCC fibers have natural colors. The utilization of NCC fiber in the fabric would minimize textile processing and extensively reduce the generation of toxic chemical wastes [1]. Nevertheless, poor fiber quality and dull colors have greatly hindered the large-scale utilization of NCC fiber [2].

Understanding of the pigmentation in NCC fiber has been largely promoted in the last two decades. The currently available NCCs mainly produce green or brown fibers. Early studies show that the flavonoids, mainly the derivatives of flavones and flavonols, play crucial roles in the coloration of green cotton fiber (GCF) [3]. Subsequent research has revealed the positive correlation between the degree of GCF color and the concentration of caffeic acid derivatives generated through the phenylpropanoid biosynthesis pathway [4]. Likewise, some other work has revealed that the proanthocyanidins (PAs) are the main pigment compositions of brown cotton fiber (BCF), with the oxidation products directly contributing to the coloration [5,6,7,8,9].

PAs (or condensed tannins) are synthesized via the phenylpropanoid—flavonoid—proanthocyanidin pathway. Key enzymes involved in PA biosynthesis, transport and regulation have been characterized in many plants [10,11,12,13]. Some independent research has revealed that the expression of several structure genes is related to the cotton fiber coloration, including phenylpropanoid biosynthetic gene cinnamic acid-4-hydroxylase (C4H) and the core flavonoid biosynthetic genes chalcone synthase (CHS), chalcone isomerase (CHI), flavanone 3-hydroxylase (F3H), flavonoid 3’-hydroxylase (F3’H) and flavonoid 3’,5’-hydroxylase (F3’5’H), as well as dihydroflavonol 4-reductase (DFR), anthocyanidin synthase (ANS) and anthocyanidin reductase (ANR), which are involved in anthocyanidin and PA biosynthesis branches [5,6,14]. More recently, Liu et al. revealed that CHI is involved in the coloration of BCF and that its inhibition would generate three fiber phenotypes (BCF, GCF and WCF) in the offspring [15].

Leucoanthocyanidin reductase (LAR) and ANR (together with ANS) function in the PA branch of flavonoid biosynthesis and convert the flavan-3,4-diol into catechin and epicatechin, respectively [16]. Interestingly, individual biochemical analyses of the monomeric composition analyses indicated different PA flows in BCFs, e.g., Feng et al. suggested that ANR played major roles in PA biosynthesis in BCFs [8], while Xiao et al. stated that LAR represented the major flow [17]. In more recent work, through the CLCrV-based virus-induced gene silencing system, Gao et al. silenced three key genes involved in PA biosynthesis (CHS, LAR and ANR) and found that all their expressions were positively related to the fiber color depth [16]. All these facts indicate that, although there is still some controversy about the main flow, both LAR and ANR function in NCC fiber coloration.

Given the somewhat unstable and monotonous nature of NCC fiber color, breeding novel lines with diverse hues is one of the primary objectives for the broader application of NCC fibers. Wild cotton species are valuable resources for improving the agricultural traits of cultivated cotton species, such as fiber quality and stress tolerance. Moreover, given the fact that most of the wild cotton species produce brown fibers, such as *G. stocksii* (E_1_E_1_)*, G. raimondii* (D_5_D_5_) and *G. longicalyx* (F_1_F_1_), they are also of great application potential in the improvement of cotton fiber color. However, almost all the current knowledge of fiber pigmentation in genus *Gossypium* is exclusively derived from the studies on *G. hirsutum* NCCs, while still little is known about that in wild cotton species.

Recently, we constructed a chromosome-level genome assembly of *G. stocksii* to gain insight into its drought-responsive mechanisms [18]. In the present work, based on the established genome annotation, we identified the PA biosynthetic genes in *G. stocksii* and comparatively analyzed their expression during fiber development against their counterparts in the cultivate diploids *G. arboreum* (A_2_A_2_). We believe this work will enhance our understanding of the mechanisms of PA biosynthesis and fiber pigmentation in *Gossypium* and also promote the application of wild cotton germplasm resources in the breeding of novel NCC accessions.

## 2. Results

### 2.1. Accumulation of PAs in Developing G. stocksii Fibers

**Figure 1:**
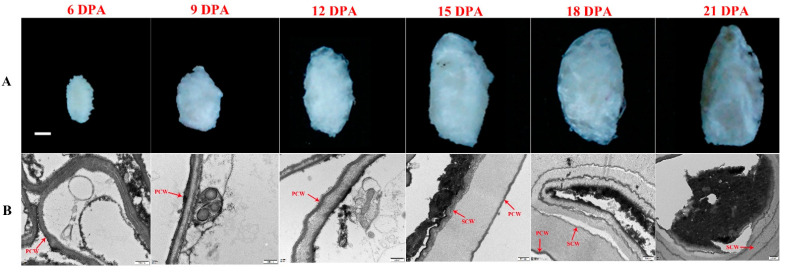
Fiber development in *Gossypium stocksii*. (**A**) Morphology and coloration of the developing fibers. Scale bar: 1 mm. (**B**) Microstructures of the developing fibers. Apparent secondary cell wall was observed at 18 DPA. PCW: primary cell wall; SCW: secondary cell wall. scale bar: 500 nm.

Development of *G. stocksii* fiber requires a shorter time period than the *G. hirsutum* NCCs (45–60 days), with the four stages, i.e., initiation, elongation, secondary cell wall (SCW) biosynthesis and maturation, spanning approximately 25–35 days. Although slight brown fuzz could be found around 12 days after anthesis (DPA), apparent brown fiber was only observed around 18 DPA, and the coloration continuously darkened until the boll opening period (Figure 1). In this work, according to the microstructures of the developing fibers, growth of primary cell wall (PCW) was observed at 6 to 12 DPA, but the SCW was only observed from 15 DPA and thickened in subsequent periods, indicating that the stage of transition from fiber elongation to SCW biosynthesis was around 12–15 DPA in *G. stocksii*.

**Figure 2:**
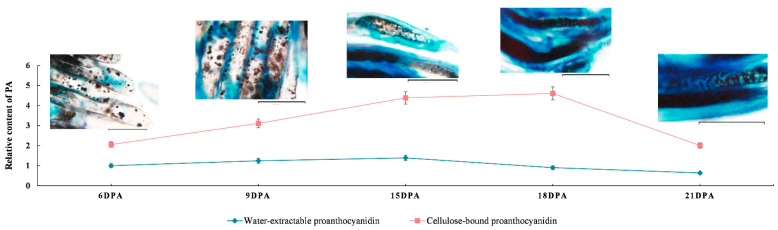
Accumulation of proanthocyanidins (PAs) during fiber development in *Gossypium stocksii* (6–21 DPA). The TBO staining was performed to observe the accumulated PAs using the fibers from the same days as colorimetrical analyses (scale bar: 20 µm).

Toluidine blue O (TBO) staining was used to assay the accumulation of PAs in developing *G. stocksii* fibers. We found that TBO stained dots scattered in the fibers at 6 and 9 DPA, indicating that the PAs had been synthesized and deposited then. At 15 and 18 DPA, the stained patches were increased in the fibers but significantly reduced at 21 DPA. This suggests that the PA synthesis was maintained at a high level during the stages of *G. stocksii* fiber elongation and early SCW biosynthesis, but was significantly reduced when SCW thickened (Figure 2).

Subsequent spectrophotometer colorimetry analyses revealed a similar dynamics process of PA accumulation in *G. stocksii* fibers. These analyses showed that the content of water-extractable PAs slightly varied during fiber development. In contrast, the content of cellulose-bound PAs significantly increased from 6 DPA, peaked around 18 DPA and decreased at 21 DPA, with the alteration up to twofold. In addition, the colorimetry analyses also showed that the content of cellulose-bound PAs was about two fold to fourfold greater than water-extractable PAs in *G. stocksii* fibers.

### 2.2. Gene Expression Variation during G. stocksii Fiber Development

To get insight into the PA biosynthesis during *G. stocksii* fiber development, we checked the gene expression variation in fibers of 6, 12 and 18 DPA using RNA sequencing. About 20 to 30 million short reads were generated from sequencing of the constructed cDNA libraries, with about 97% mapped to the reference genome (Table S1).

**Figure 3:**
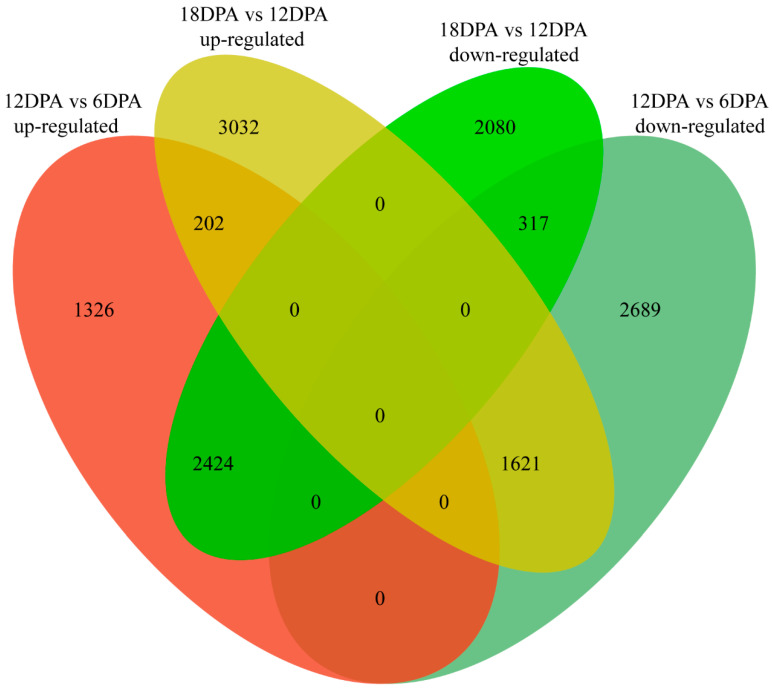
Statistics of differentially expressed genes between the *Gossypium stocksii* fibers of 6, 12 and 18 DPA.

Comparative analyses identified 8579 and 9676 differentially expressed genes (DEGs) between the fibers of 12 and 6 DPA, as well as 18 and 12 DPA, respectively (hereafter referred to as DEGs of 12v6 and 18v12, respectively), representing about 20% of the whole set of protein-coding genes (Figure 3). Interestingly, we found that many genes (about 5% of the entire gene set) were regulated in different manners from 6 to 12 DPA, and from 12 to 18 DPA. For example, 23 tubulin subunit encoded genes were upregulated at 12 DPA as compared to 6 DPA, but 21 were then downregulated at 18 DPA. Likewise, genes coding for AP complexes, coatomer subunits, and several vesicle-associated membrane proteins were regulated similarly, e.g., during *G. stocksii* fiber development, 44 related genes were upregulated at 12 DPA, while 36 were subsequently downregulated at 18 DPA. All these findings showed large-scale gene expression modulation during *G. stocksii* fiber development and indicated the variation in major physiological features between the stages of fiber elongation and SCW biosynthesis.

**Figure 4:**
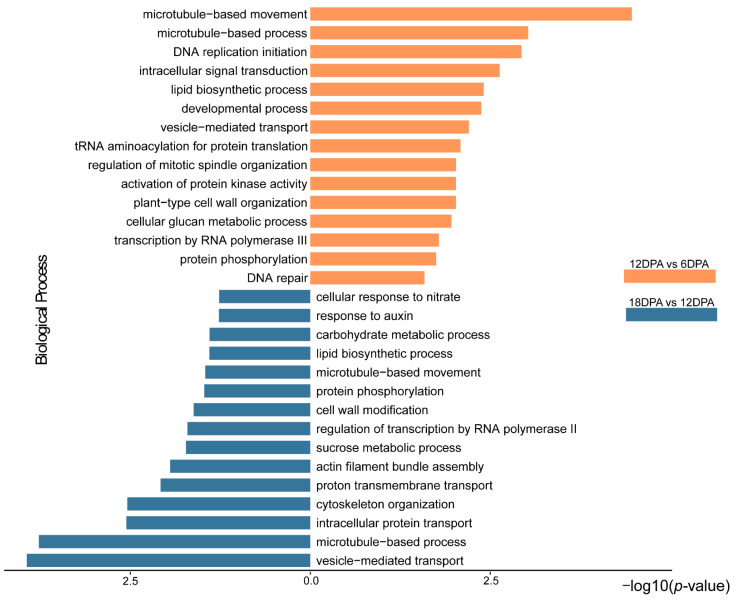
Gene ontology enrichment analyses of the genes differentially expressed between *Gossypium stocksii* fibers of 6, 12 and 18 DPA.

**Figure 5:**
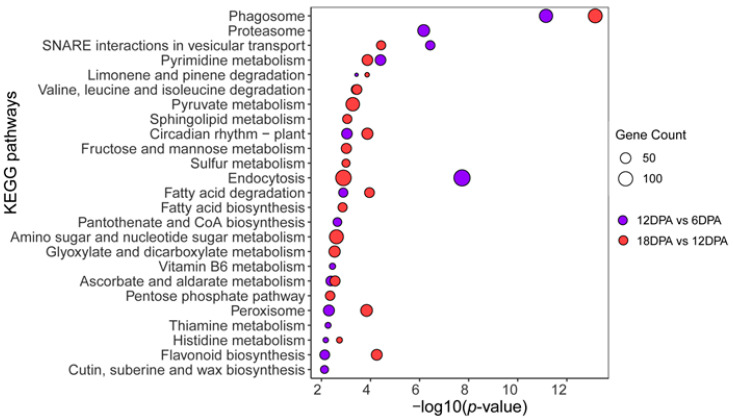
KEGG pathway enrichment analyses of the genes differentially expressed between *Gossypium stocksii* fibers of 6, 12 and 18 DPA.

Functional analyses showed that both DEG collections were mainly enriched in the biological processes associated with the fiber development and growth, such as ‘microtubule-based process (GO:0007017)’, ‘vesicle-mediated transport (GO:0016192)’, ‘cytoskeleton organization (GO:0007010)’, and ‘cell wall modification (GO:0042545)’ (Figure 4). In addition, pathway enrichment analyses showed the extensive expression variation of genes related to the phagosome, vesicular transport, and endocytosis (Figure 5), possibly consistent with the enhanced cell wall morphogenesis during fiber development [19]. Notably, according to the observed variation in PA content in developing fibers, we also found the enrichment of flavonoid biosynthesis-related genes in both DEG collections of 12v6 and 18v12, referring to most of the components involved in flavonoids and PA biosynthesis.

### 2.3. Identification and Comparative Analyses of PA Biosynthetic Genes in Cotton Species

For more detailed analyses of the molecular mechanisms of PA biosynthesis in *G. stocksii* fiber, we tried to identify and compare the related gene components in the diploid cotton species with well-annotated genomes. Based on searching orthologs characterized in *Arabidopsis thaliana*, structural genes of the PA biosynthesis pathway were identified in *G. stocksii* (E-genome), *G. raimondii* (D-genome, BCF) and *G. arboreum* (A-genome, WCF) (Table S2). As LAR is absent in *A. thaliana*, the *G. stocksii* LAR genes were identified based on the homology search using annotated LARs in *G. hirsutum*. Comparative analyses revealed that the orthologs of PA biosynthetic genes were highly conserved in primary sequence among the investigated *Gossypium* species, with almost all the amino acid identities >95%.

A series of transcription factors (TFs) are involved in regulating plant anthocyanins and PA biosynthesis. A homology search against the predicted proteome of *G. stocksii* identified several orthologs of characterized regulators in *A. thaliana*. Similar to the structural genes, these TFs were also conserved between *G. stocksii*, *G. arboreum* and *G. raimondii*, with the identities ranging from 88% to 99%. These facts indicated that the structural genes and regulators of the PA biosynthesis pathway were highly conserved in biological function in cotton species producing either BCF or WCF.

### 2.4. Expression Analyses of PA Biosynthetic Genes in Developing G. stocksii Fibers

**Figure 6:**
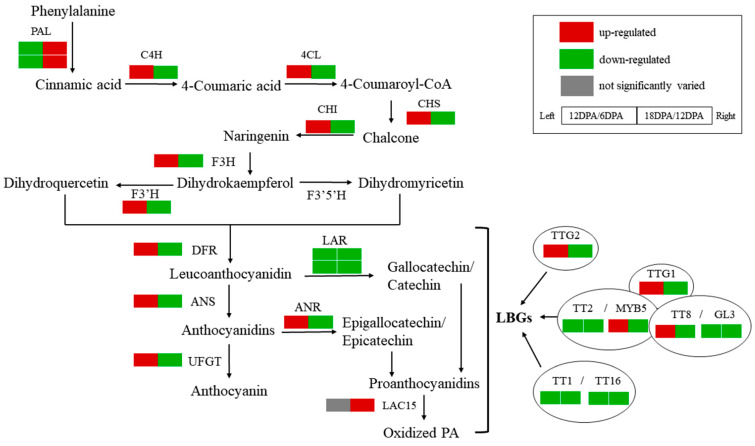
Differentially expressed proanthocyanidin biosynthetic genes during fiber development in *Gossypium stocksii* (6, 12, and 18 DPA). LBGs: late biosynthetic genes.

Comparative transcriptomics analyses revealed that the phenylpropanoid biosynthesis was downregulated along with the *G. stocksii* fiber development (Figure 6 and Table S3). In contrast, the flavonoid biosynthesis was enhanced at 12 DPA as compared to 6 DPA, with the expression of CHS, F3H, DFR and ANS significantly upregulated. Nevertheless, all the flavonoid biosynthetic genes were dramatically downregulated at 18 DPA (log2foldchange ranged from −4 to −11).

For the genes involved in the PA branch, LAR genes were downregulated during the fiber development, with the accumulation of LAR1 transcript sharply reduced at 18 DPA. Unlike LARs, the ANR was upregulated at 12 DPA compared to 6 DPA and expressed at a much higher level than LARs, about 20 and 150 times at 6 DPA and 12 DPA, respectively (Table S3).

Involvement of plant polyphenol oxidases (PPOs) and laccases (TT10) in the subsequent oxidative polymerization of PAs have been previously discussed [8,20]. However, for the 12 PPOs encoded by *G. stocksii* genome, 11 were not expressed in fibers according to our RNA-seq data, while expression of the other one (GS10G_19100) was only detected at 6 DPA. In contrast, LAC15 (GS09G_28530), the *G. stocksii* homolog of *A. thaliana* TT10, was not detectable in the early stages (6 DPA and 12 DPA) but was remarkably accumulated at 18 DPA.

For the PA transporters, GSTF12 was lowly expressed along with the fiber development (FPKM < 1), in contrast to TT12 and AHA10, which were expressed at a much higher level. Moreover, the expression of TT12 was not significantly altered at 12 DPA as compared to 6 DPA, whereas AHA10 was significantly upregulated. Both TT12 and AHA10 were downregulated at 18 DPA as compared to 12 DPA.

Expression of the regulators involved in plant PA biosynthesis was investigated in developing *G. stocksii* fiber as well. TT2, GL3, MYC1, TT1 and KAN4 were almost specifically expressed in the early period of fiber elongation. WER, MYB61/5, TT8 and TTG2 were upregulated during fiber elongation (12 DPA versus 6 DPA), with WER and TT8 being downregulated in the early SCW biosynthesis stage (18 DPA).

**Figure 7:**
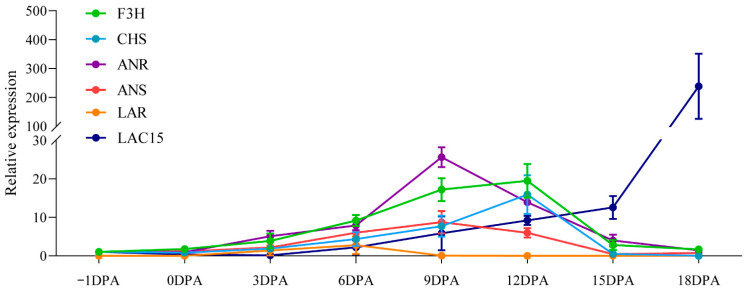
qRT-PCR analyses of proanthocyanidin biosynthetic genes in developing *Gossypium stocksii* fibers (−1 to 18 DPA).

qPCR analyses were performed to validate the expression of PA biosynthetic genes in developing *G. stocksii* fibers. Consistent with the transcriptomics analyses, transcripts levels of CHS, F3H, ANS and ANR peaked around 9 to 12 DPA (Figure 7). Interestingly, the transcript’s accumulation of ANS and ANR peaked earlier than that of CHS and F3H. qPCR analyses also indicated that the expression level of LARs was significantly lower than ANRs in *G. stocksii* fiber. During the period from 6 to 9 DPA, ANR transcript was remarkably accumulated, but the level of LARs transcript was decreased to an extremely low level.

### 2.5. Variation of Gene Expression Modulation in Developing Fibers between G. stocksii and G. arboreum

**Figure 8:**
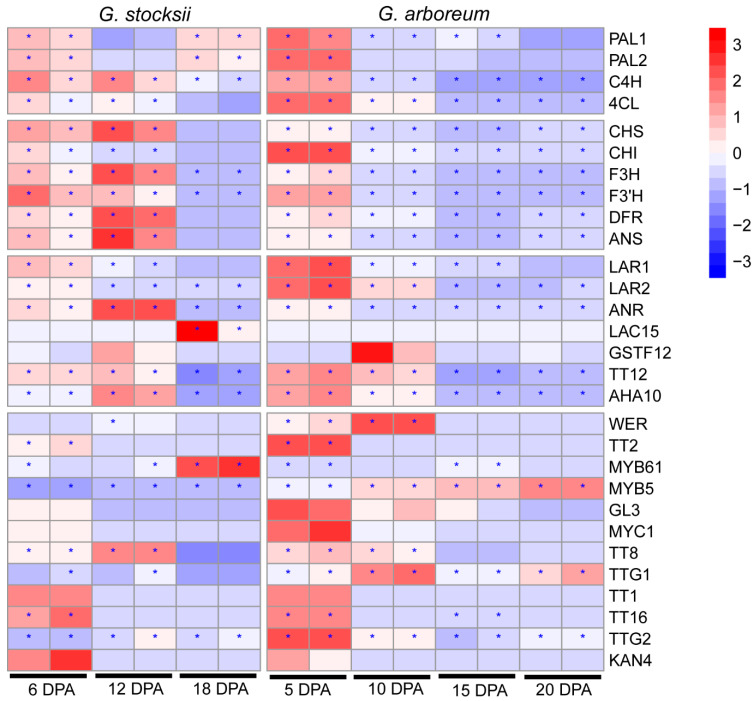
Expression of proanthocyanidin biosynthetic genes in developing fibers of *Gossypium stocksii* and *Gossypium arboreum*. The filled colors indicate the gene expression levels (from low: blue to high: red) (scaled by raw). The asterisks indicate the expression levels of FPKM > 10.

To further understand the molecular mechanisms of fiber coloration in cotton species, we also investigated the expression of PA biosynthetic genes in *G. arboreum*, another diploid cotton species that produces white fiber. We found that starting from the early period of fiber elongation (10 DPA), almost all the structural genes involved in the phenylpropanoid-flavonoid-proanthocyanidin pathway were downregulated in *G. arboreum*, in contrast to some genes like F3H and CHS, which were still upregulated in *G. stocksii (*Figure 8*)*. Notably, during stage transition from fiber elongation to SCW biosynthesis, i.e., around 12–15 DPA in *G. stocksii* and 15–20 DPA in *G. arboreum*, these PA biosynthetic genes were remarkably more highly expressed in the brown fiber of *G. stocksii*. For instance, transcriptomics analyses showed that the accumulation of CHS, F3H, DFR, ANS and ANR transcripts was about 20-fold higher in *G. stocksii* (12 DPA) than *G. arboreum* (15 DPA). Meanwhile, expression of the potential PA transporter AHA10 was not altered in *G. arboreum* during the early days of SCW biosynthesis (15 DPA), in contrast to its significant upregulation in *G. stocksii.*

Besides, expression modulation of the regulatory components in developing fibers was also largely varied between *G. stocksii* and *G. arboreum* during elongation–SCW biosynthesis transition, e.g., WER and TT8 were upregulated in *G. stocksii* but were downregulated in *G. arboreum*. In contrast, TT16 was downregulated in *G. stocksii* but was up-regulated in *G. arboreum*. Interestingly, during fiber development of both cotton species, e.g., from 6 DPA to 18 DPA in *G. stocksii* and from 5 DPA to 20 DPA in *G. arboreum*, expression of TT2 genes was continuously downregulated.

## 3. Discussion

Despite the growing attention to NCC fiber in recent years for its ecologically friendly nature, some issues remain before its large-scale application in the textile industry can be achieved. One of the most important is how to breed novel NCC lines that produce fibers with more varied and stable colors. Due to the limited genetic background of current NCCs, utilization of germplasm resources from wild cotton species, especially those producing brown fibers, may be one resolution to break the bottleneck of NCC breeding. Meanwhile, exploration of the molecular mechanisms of fiber pigmentation in a broader range of cotton species would promote our understanding of the NCC fiber coloration and provide new strategies for improving the NCC fiber color by genetic engineering methods. *G. stocksii* produces a brown fiber with high strength and is tolerant to various environmental stresses, thus highlighting its value in cotton breeding. However, before further application, mechanisms of its fiber coloration and the association between the fiber color and quality still need to be explored.

Feng et al. revealed that the accumulation of PAs in the brown fiber of NCCs (*G. hirsutum*) started in the early days of fiber development and peaked around 30 DPA, then the total PAs were decreased due to their oxidization [8]. In this work, we observed that the accumulation of PAs in the *G. stocksii* fiber started from 6 DPA or earlier, peaked around 15 DPA, and decreased at 21 DPA. This indicated that the accumulation of PAs in brown fibers was very similar, i.e., the PA biosynthesis started from the early days after anthesis, peaked at the first days of SCW biosynthesis, and decreased during maturation.

LAR and ANR are the key enzymes of the PA branch. Although it is controversial as to which is more important in cotton fiber coloration, involvement of both was recently revealed [16]. Nevertheless, derived from the expression analyses in developing *G. stocksii* fibers, the LARs were downregulated at 12 DPA compared to 6 DPA, whereas the ANR was significantly upregulated and expressed at a much higher level, indicating the ANR might also represent the major flow in PA biosynthesis in *G. stocksii* fiber.

Notably, although the PA content peaked around 15 DPA in *G. stocksii*, the apparent fiber color was observed about 18 DPA, suggesting that, similarly to the *G. hirsutum* NCC accessions, it was possibly oxidized derivatives of PAs that directly conferred the fiber coloration. Until recently, the exact enzymes involved in PA oxidation in cotton fibers remained unclear. The involvement of PPOs was discussed previously [8], but as revealed by the RNA-seq data in this work, expression levels of almost all the PPO genes were not detectable in developing fibers of *G. stocksii*, suggesting other enzymes might be required for the PA oxidation and fiber coloration. Meanwhile, involvement of LAC15 in the oxidative polymerization of flavonoids has been revealed in *A. thaliana* and was discussed in some other plants, such as *Brassica napus* and *Litchi chinensis* [21,22,23]. In this work, high temporal specificity of LAC15 expression in *G. stocksii* fiber strongly indicated its association with the fiber development. However, whether it is indeed required for the PA oxidation and further oligomerization still needs future exploration.

In addition, we found that for the three candidate PA transporters, expression of GSTF12 was almost undetectable in *G. stocksii* fiber and expression of TT12 was not regulated along with the PA accumulation, while the AHA10 was expressed at a high level and significantly upregulated during fiber elongation. This suggests that AHA10 was probably the main PA transporters in developing *G. stocksii* fiber. Unfortunately, the involvement of AHA in PA accumulation in the vacuolar was exclusively characterized in *A. thaliana*, thus, whether it plays similar roles in a wider range of plants still needs further investigation.

The primary sequences of PA biosynthetic genes were highly conserved between cotton species, either producing brown (*G. raimondii*) or white (*G. arboreum*) fiber. Therefore, this indicates that the difference in fiber coloration might be due to other factors rather than the functional deviation of these gene components. Previous work revealed that the expression levels of PA biosynthetic genes, such as CHI, F3H, DFR, ANS and ANR, were much higher in color cottons [5]. In this work, in addition to significantly higher expression levels, we found upregulation of these genes through extended fiber developmental stages in *G. stocksii*, i.e.*,* they were downregulated in the elongation stage (10 DPA) in *G. arboreum *but still upregulated in *G. stocksii* (12 DPA). Thus, the higher expression level during fiber development and the continuous enhancement of gene expression during fiber elongation might be important for PA accumulation in *G. stocksii* fiber. Actually, a similar variation has been found previously when Li et al. investigate the pigmentation in brown fiber of NCCs, i.e., they revealed that PA content peaked at 5 DPA in the white fiber, but was increased from 5 to 15 DPA in brown fiber [6].

Consistent with the different expression of PA biosynthetic genes in developing fibers of *G. stocksii* and *G. arboreum* during late fiber elongation and early SWC biosynthesis, the expression of the potential regulators was broadly varied. In *A. thaliana*, PA synthesis is mainly regulated by the MBW complex TT2-TT8-TTG1, while the other three complexes are also involved, such as MYB5-TT8-TTG1, TT2-EGL3-TTG1 and TT2-GL3-TTG1 [24]. In cotton species, the biological role of TT2 in the pigmentation of BCF has been revealed in independent research. Hinchliffe et al. revealed that activation of the TT2 gene (GhTT2_A07) would upregulate the genes involved in phenylpropanoid and flavonoid pathways [25]. In addition, Yan et al. showed that TT2 (GhTT2-3A) could activate TT8 and then enhance the PA biosynthesis and accumulation in cotton fibers [26]. Unexpectedly, although the accumulation of PAs in *G. stocksii* fibers significantly increased during stages of fiber elongation, TT2 was expressed at a low level then and almost undetectable during SCW thickening. On the contrary, TT8 was highly expressed, and its expression was regulated in the same manner with the structural genes, i.e., upregulated at 12 DPA and downregulated at 18 DPA. This was different from *G. arboreum*, where the expression of TT8 was downregulated in the late fiber elongation stage. These facts collectively suggest that it might be the TT8 that is mainly responsible for the synthesis of PAs in *G. stocksii* fiber. Meanwhile, we noticed that the TFs WER and MYB5 were also upregulated during fiber elongation in *G. stocksii* fiber compared to the downregulation of their counterparts in *G. arboreum*. Therefore, it is possible that, rather than TT2-TT8-TTG1, the other complex, like MYB5-TT8-TTG1, is the main regulatory complex of PA biosynthesis in developing *G. stocksii* fibers.

## 4. Materials and Methods

### 4.1. Plant Materials

The *G. stocksii* plants were grown at the Laboratory of Plant Genome and Colored Fiber Molecular Improvement, College of Life Sciences and Medicine, Zhejiang Sci-Tech University, Hangzhou, China.

### 4.2. Microstructure Observation and Histochemical Staining

The fresh *G. stocksii* fibers from 6, 9, 12, 15, 18 and 21 DPA were fixed with 2.5% glutaraldehyde in 0.1 M phosphate buffer (pH 7.0) for more than 4 h, and washed three times in the phosphate buffer for 15 min at each step. Then, they were postfixed with 1% OsO_4_ in phosphate buffer for 1 h and washed three times in the phosphate buffer for 15 min at each step. Subsequently, samples were dehydrated by a graded series of ethanol (30%, 50%, 70% and 80%) and acetone (90% and 95%) for about 15 min at each step. Next, the samples were dehydrated twice by absolute acetone for 20 min. The specimens were placed in a 1:1 mixture of absolute acetone and the final Spurr resin mixture for 1 h at room temperature, then transferred to a 1:3 mixture of absolute acetone and the final resin mixture for 3 h and final Spurr resin mixture overnight. Then, the specimens were placed in eppendorf containing Spurr resin and heated at 70 °C for more than 9 h. The specimens were sectioned in LKB 11800 PYRAMITOME (LKB-BROMMA, Stockholm, Sweden) and the sections were stained with 0.5% of toluidine blue O (TBO). The staining results were observed and documented under the microscope Olympus IX73 (Olympus, Tokyo, Japan). The remaining specimens were sectioned in LEICA EM UC7 ultratome (Leica Microsystems, Heidelberg, Germany) and the sections were stained by uranyl acetate and alkaline lead citrate for 5 to 10 min, respectively, and observed in a Hitachi Model H-7650 TEM (Hitachi High-Technologies, Ibaraki, Japan).

### 4.3. Analyses of PA Content in G. stocksii Fibers

The procyanidin B2 (CAS NO: 29106-49-8) was used for the preparation of the standard curve. The standard was diluted into five concentrations (0.1, 0.25, 0.5, 0.7 and 1 mg/mL) and the absorbance values at 550 nm were measured by spectrophotometer (UV-2600, Shimadzu, Japan) to plot the standard curve. Cotton fibers were collected at 6, 9, 15, 18 and 21 DPA. PA extraction was mainly performed according to the previously described methods [8,27]. In short, 50 mg fibers were cut and placed in a centrifuge tube, and 25 mL of 70% acetone solution was added overnight. The supernatant was used to estimate the content of water-extractable PAs and the sediment was washed with 3 mL SDS-2-mercaptoethanol solution (pH 8.0) into a 50 mL centrifuge tube. Next, 30 mL of n-butanol monohydrochloric acid solution was added. The tube was then placed in a boiling water bath for 75 min and cooled on ice and the supernatant was used to assay the content of cellulose-bound PAs. The solution concentrations of both water-soluble and cellulose-bound PAs were determined colorimetrically at 550 nm. The relative PA contents in individual samples were estimated by comparing to water-soluble PA content at 6 DPA.

### 4.4. Identification of PA Biosynthetic Genes in Gossypium Species

PA biosynthetic genes and the related transcription factors in *Arabiodopsis thaliana* were collected according to the genome annotation (TAIR10, arabidopsis.org) and a previously summarized gene list [28]. LAR genes Gh_A12G1558 and Gh_D12G2642 were collected from *G. hirsutum* TM-1 genome annotation (NAU assembly) [29]. BLASTP (cutoff E-value 1 × 10^−8^) was then used to identify their orthologs in available genome annotations of *G. arboreum* and *G. raimondii* through the webserver provided by CottonGen [30], and in the *G. stocksii* assembly GCA_020496765.1 that is deposited in the website of National Center for Biotechnology Information (NCBI) [18].

### 4.5. RNA Extraction, cDNA Library Construction and RNA Sequencing

Tissue samples were collected from ovules at 6 DPA and fibers at 12 and 18 DPA. The samples were frozen immediately in liquid nitrogen and stored at −80 °C until use. Total RNA was extracted using RNAprep Pure Plant kit (TIANGEN BIOTECH, Beijing, China) and treated with RNase-free DNase I. cDNA libraries were constructed according to the methods described previously [31]. RNA sequencing (RNA-seq) was performed on Illumina NovaSeq platform with a 150 bp pair-end sequencing strategy.

### 4.6. Identification of Differentially Expressed Genes (DEGs)

For quality control of the short reads generated in this work, adapter trimming and low-quality read filtering was first carried out by using the software package FASTX_Toolkit (http://hannonlab.cshl.edu/fastx_toolkit/, accessed on 1 April 2021). HISAT2 (version 2.2.1) was used to map the clean data to the *G. stocksii* genome sequence [18,32], with the read counts for coding genes estimated using HTSeq-count (version 0.11.2) [33]. DEGs between groups were determined using DESeq2 (version 1.30.1), with the cutoff fold change ≥2 and adjusted *p*-value ≤ 0.05 [34]. The gene expression levels were calculated using fragments per kilobase of transcript per million fragments (FPKM) by the StringTie (version 2.0.6) [35]. Gene ontology and KEGG pathway enrichment analyses were performed using the R package clusterProfiler (version 3.14) [36].

RNA-seq data of the developing *G. arboreum* fibers were retrieved from SRA, including SRR9695831-SRR9695836 for fibers at 5, 10 and 20 DPA, as well as SRR10609483-SRR10609484 for 15 DPA. The short reads were mapped to the reference genome (*G. arboreum*, ‘SXY1’ genome WHU-updated v1), and the DEGs were identified according to the strategy described above [37].

### 4.7. Quantitative Real-Time PCR Analyses

Ovules of −1, 0, 3 and 6 DPA and fibers of 9, 12, 15 and 18 DPA were obtained from *G. stocksii* plants. The samples were frozen immediately in liquid nitrogen and stored at −80 °C until use. Expression of six genes (CHS, F3H, ANS, LAR, ANR and LAC15) in the selected samples were analyzed by qRT-PCR (Table S4). The qRT-PCR assay was performed using NovoStart SYBR qPCR SuperMix Plus (Novoprotein Scientific Inc., Shanghai, China) with ABI QS3 fluorescence quantitative PCR instrument (ABI, CA, USA). PCR amplification employed a 10 s denaturing step at 95 °C, followed by 15 s at 95 °C and 1 min at 58 °C with 40 cycles. Relative mRNA levels were calculated by the 2^−ΔΔCt^ method with GhUBQ7 (accession number: DQ116441) as an internal control.

## 5. Conclusions

Wild cotton species account for most of the genus *Gossypium* and are valuable reservoirs for germplasm improvement of cultivated species. This work showed that PAs and derivatives contributed to the pigmentation in *G. stocksii* fiber and revealed the extensive variation of expression and regulation of PA biosynthetic genes between *G. stocksii* and *G. arboreum*. We believe it will enhance our understanding of the molecular mechanisms of PA biosynthesis in plants and provide new ideas and pathways for the improvement of NCC fiber.

## Data Availability

All the RNA-seq data generated and used in this work have been deposited in Sequence Read Archive (SRA) of the NCBI under the accession numbers SRR16573626 —SRR16573631.

## Supplementary Materials

The following are available online at www.mdpi.com/article/10.3390/ijms23021008/s1.
